# Supplementary material for: Association between serum ferritin and uric acid levels and nonalcoholic fatty liver disease in the Chinese population
Source: PeerJ. 2023 Oct 26;11:e16267. doi: 10.7717/peerj.16267 (PMC10613435; doi:10.7717/peerj.16267)
Supplement: Supplemental Information 2 [file peerj-11-16267-s002.docx]

**Present history:** 0 indicate no disease; 1 indicate present other diseases;

**Past history:** 0 indicate No; 1 indicate Yes;

**Family history:** 0 indicate No; 1 indicate Yes;

**History of infectious diseases:** 0 indicate No; 1 indicate Yes;

**History of smoking:** 0 indicate No smoking;

**History of alcohol consumption:** 0 indicate No alcohol consumption;
